# The Distribution and Host-Association of the Vector Chigger Species *Leptotrombidium imphalum* in Southwest China

**DOI:** 10.3390/insects15070504

**Published:** 2024-07-05

**Authors:** Qiao-Yi Liu, Rong Fan, Wen-Yu Song, Pei-Ying Peng, Ya-Fei Zhao, Dao-Chao Jin, Xian-Guo Guo

**Affiliations:** 1 Institute of Pathogens and Vectors, Yunnan Provincial Key Laboratory for Zoonosis Control and Prevention, Dali University, Dali 671000, China; 2 Institute of Microbiology, Qujing Medical College, Qujing 655100, China; 3 Institute of Entomology, Guizhou University, Guiyang 550025, China

**Keywords:** chigger mite, *Leptotrombidium imphalum*, vector of scrub typhus, southwest China

## Abstract

**Simple Summary:**

*Leptotrombidium imphalum* (a chigger species) can serve as a transmitting vector of scrub typhus. Based on the field investigation in southwest China from 2001 to 2022, this article presents the first report on the distribution and infestation of *L. imphalum* on small mammals in the region. A total of 2161 *L. imphalum* were identified from 218 small mammal hosts that crossed three orders, and the majority of the mites were found on the order Rodentia (rodents). Different host species had different susceptibility to the infestation of *L. imphalum*, and the shrew gymnure (*Neotetracus sinensis*) was much more susceptible to the infestation than other host species. *Leptotrombidium imphalum* exhibited an aggregated distribution among different individuals of its hosts. The positive correlation between the infestation indices for *L. imphalum* on small mammals and the occurrence of scrub typhus, together with the low host specificity of the mite, indicates the potential risk of the mite.

**Abstract:**

*Leptotrombidium imphalum* is a species of chigger mites, and it can serve as a transmitting vector of scrub typhus. Southwest China is an important focus of scrub typhus. Based on the field investigation in southwest China from 2001 to 2022, this article presents the first report on the distribution and infestation of *L. imphalum* on rodents and other sympatric small mammals in the region. A total of 2161 *L. imphalum* were identified from 218 small mammal hosts in 21 of 114 survey sites. The 17 host species of *L. imphalum* crossed 13 genera and 5 families in 3 orders (Rodentia, Eulipotyphla, and Scandentia), indicating the low host specificity of the mite. The Asian house rat (*Rattus tanezumi*) was the dominant host species in the 21 sites where *L. imphalum* were collected, and 49.38% of mites were found on *R. tanezumi*. Different small mammals had different susceptibility to the infestation of *L. imphalum*. The prevalence (*P_M_* = 27.66%), infestation mean abundance (*MA* = 6 mites/per examined host), and mean intensity (*MI* = 21.69 mites/per infested host) for *L. imphalum* on the shrew gymnure (*Neotetracus sinensis*) were much higher than those on other host species (*p* < 0.05), indicating *N. sinensis* had a high susceptibility to the infestation of *L. imphalum*. The infestation indices for *L. imphalum* on small mammal hosts varied along different altitude and latitude gradients (*p* < 0.05), indicating the environmental heterogeneity of the mite infestation. *Leptotrombidium imphalum* exhibited an aggregated distribution among different individuals of its hosts. Besides the low host specificity of *L. imphalum*, the prevalence of the mite was positively correlated with the occurrence of scrub typhus, indicating the potential risk of the mite.

## 1. Introduction

Chigger mites (or chiggers) are a group of tiny arthropods, and they are the exclusive transmitting vector of scrub typhus (tsutsugamushi disease). Most chigger species are ectoparasites of other animals (vertebrates, invertebrates, and even some arthropods), especially rodents and other sympatric small mammals [1,2,3,4]. As the main hosts of chiggers, rodents and other small mammals are the main infection source and reservoir host of *Orientia tsutusgamushi*, the pathogen of scrub typhus. Through the biting activity of chiggers, *O. tsutusgamushi* (Ot) can be transmitted from rodents to humans [5,6]. Besides transmitting Ot, some chigger species (e.g., *Leptotrombidium scutellare* Nagayo et al., 1921) can serve as the potential vector of *Hantavirus* (HV), the pathogen of hemorrhagic fever with renal syndrome (HFRS) [7,8]. Southwest China (21°08′–33°41′ N, 97°21′–110°11′ E) is a vast territory, including five provincial regions, Yunnan, Guizhou, Sichuan, Chongqing, and Tibet (Xizang Autonomous Region). Scrub typhus and HFRS are prevalent in southwest China, with some local areas experiencing an outbreak, and it is of medical significance to study chiggers in the region [9,10,11]. In addition, there are Yunnan-Guizhou Plateau, Sichuan Basins, Tibetan Plateau, and Hengduan mountains within the territory of southwest China, with diverse topography, different geographical landscapes, and different types of vegetation and climates, which provide an ideal place to study the distribution and ecological issues of chigger mites on small mammals [12,13].

Although more than 3000 chigger species have been documented in the world, only a few dozens of species (around 50 species) have proved to be the effective vectors of scrub typhus, and 10 of them are the powerful vectors of the disease [6,14,15,16,17,18]. So far, the confirmed vectors of scrub typhus are mainly concentrated in the genus *Leptotrombidium*, which has the greatest medical significance [6,14,19,20,21,22]. *Leptotrombidium imphalum* was described and named by Vercammen-Grandjean and Langston in 1975, and it is one of valid species in the genus *Leptotrombidium* [23,24]. In some foci of scrub typhus, *L. imphalum* is one of important vectors of scrub typhus, and for example, it has been confirmed as one of the vectors of this disease in Thailand [25,26,27,28,29]. In China, *L. imphalum* is also considered a significant potential vector of scrub typhus, and *O. tsutusgamushi* was once isolated from *L. imphalum* in Yunnan Province of southwest China [30,31].

Previous studies on *L. imphalum* mainly focused on its efficiency of transmitting the scrub typhus pathogen, *O. tsutsugamushi*, including the natural and experimental infection of Ot in *L. imphalum* as well as the transovarial and transstadial transmission of Ot in the mite [25,27,28,32,33,34,35,36,37]. Additionally, some previous studies examined the distribution of Ot in *L. imphalum* [38], the weight loss of the mouse host (*Mus musculus*) fed upon by the mite infected with Ot [39], the effective rodent hosts of the mite [25,32], and some other relevant issues [40]. To date, few reports have involved the distribution and infestation of *L. imphalum* on small mammals in a specific geographical region. From field investigations in southwest China between 2001 and 2022, we collected and identified a lot of *L. imphalum*, which aroused us to study the distribution and infestation status of this vector species on its small mammal hosts in the region. Being a retrospective study on the basis of previous field investigations, the present study aims to provide more scientific information for further research on this vector species of scrub typhus and to provide a scientific reference for the surveillance and control of the disease and its transmitting vectors in southwest China.

## 2. Materials and Methods

### 2.1. Survey Sites

**Figure 1:**
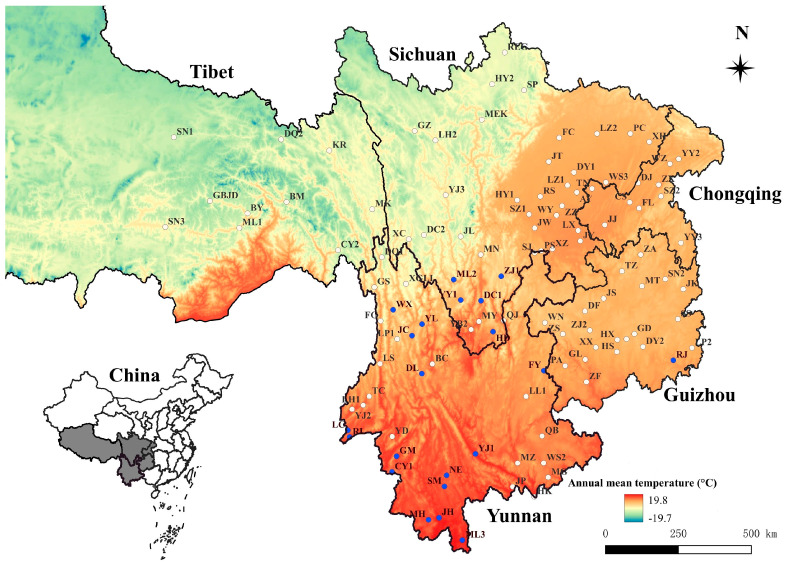
The distribution of 114 survey sites and the sites where *Leptotrombidium imphalum* was collected in southwest China (2001–2022). Annotation: The survey sites marked with blue dots were the sites where *Leptotrombidium imphalum* was collected.

The present study is a retrospective study, and its original data came from previous field investigations in 114 survey sites of southwest China between 2001 and 2022 (see “Table 1” and “Figure 1” in “Section 3”). The field investigations were carried out in different latitudes, longitudes, altitudes, landscapes (mountainous and flatland landscapes), and habitats (indoor and outdoor habitats), and the 114 survey sites are distributed in the five provincial regions of southwest China, Yunnan, Guizhou, Sichuan, Chongqing, and Tibet (Xizang Autonomous Region), covering the most territory of southwest China except western Tibet. In Tibet, only the eastern part was investigated due to the fact that the western part of Tibet is a vast and sparsely populated territory with relatively inconvenient transportation, the existence of hypoxia, and some potential risks in the sparsely populated high-cold areas, and in addition, we did not have enough human resources and financial support to cover the whole territory of Tibet.

### 2.2. Collection and Identification of Chiggers

According to the “transect line method”, cage traps (18 × 12 × 9 cm; Guixi Mousetrap Apparatus Factory, Guixi, Jiangxi, China) with fresh peanuts, corn, or other baits were placed at each survey site for capturing rodents and other sympatric small mammal hosts (shrews, tree shrews, etc.) [41]. In dry lands, every 25 cage traps (mouse traps) in a group were placed in a straight line, with a spacing of 5 m and a row spacing of 20 m. Considering the complexity and diversity of environmental conditions in the actual investigations, the placement ways of cage traps were flexibly adjusted according to the specific environmental conditions. For example, in the indoors, a cage trap was placed every 15 square meters (15 m^2^) along the base of the wall. In a paddy field, cage traps were placed along the bank of the field. The same number of cage traps should be placed at each survey site to ensure the “homogeneity” and “comparability” of sampling methods at different survey sites. Each small mammal host captured was separately placed in a cloth bag and transported to the field temporary laboratory [41,42,43,44,45]. In the temporary laboratory, each animal host was separately placed in a big white square plate to collect its ectoparasitic chiggers. Chiggers are very tiny, and they often attach themselves to thin and tender sites of the host skin, including the auricle, outer opening of external auditory canal, groin, perianal area, etc. [46,47]. In order to collect as many chiggers as possible and to ensure that the numbers of chiggers collected from every animal host are comparable, the thin and tender skin sites, especially the auricle and outer opening of external auditory canal where chiggers frequently attach, were chosen as fixed collection sites. Chiggers are very tiny, and it is very difficult to identify them with naked eyes. Under the help of a magnifier, a lancet or curette (ear scraper) was used to scrape the chiggers and chigger-sized objects (suspected chiggers) from the skin of each animal host. The collected chiggers and chigger-sized objects were preserved in 70% ethanol. In the laboratory, the chiggers and chigger-sized objects preserved in 70% ethanol were transferred into distilled water to rinse 2–3 times, and the chiggers were separated from other non-chigger debris under a stereomicroscope (Beijing Electronic Optical Equipment Factory, Beijing, China). The separated chiggers were then mounted on glass slides with Hoyer’s solution [48,49,50]. After dehydration, drying, and transparent process, each glass slide specimen of chigger was carefully observed and measured one by one under a light microscope (Olympus Corporation, Tokyo, Japan) for taxonomic identification [51,52,53,54]. After finishing the identification of all the chigger specimens, *L. imphalum* was selected as the target of the present study. The use of animals (including animal euthanasia) for our research was officially approved by the Animals’ Ethics Committee of Dali University, and the representative specimens were deposited in the specimen repository of Institute of Pathogens and Vectors, Dali University.

### 2.3. Statistics for Chigger Infestation

The constituent ratio (*C_r_*, %) was conventionally used to calculate the percentage of *L. imphalum* in the chigger community. The constituent ratio (the composition ratio, *C_r_*) is a commonly used index in statistics and reflects the proportion of an internal component in the whole, and it is usually expressed as a percentage. The total sum of the constituent ratios must be 100% [55,56,57]. The prevalence (*P_M_*, %) was used to calculate the infestation frequency of small mammal hosts with *L. imphalum*, the percentage of infested hosts. The mean abundance (*MA*) was used to calculate the average infestation intensity of *L. imphalum* on the examined hosts (chiggers/per examined host), and mean intensity (*MI*) was used to calculate the average infestation intensity of *L. imphalum* on the infested hosts (chiggers/per infested host). The formulae for *C_r_*, *P_M_*, *MA* and *MI* were as follows [55,56,57,58,59].

(1) $$C_{r}=\frac{N_{i}}{N}\times 100\%$$

(2) $$P_{M}=\frac{H_{i}}{H}\times 100\%$$

(3) $$MA=\frac{N_{i}}{H}$$

(4) $$MI=\frac{N_{i}}{H_{i}}$$

In the above formulas, *N_i_* represents the number of *L. imphalum*, *N* represents the total number of all chiggers, *H_i_* represents the number of hosts infected with *L. imphalum*, and *H* represents the total number of hosts.

### 2.4. Measurement of Spatial Distribution Pattern of L. imphalum

The Iwao’s regression model and Taylor’s power law were used to measure the spatial distribution pattern of *L. imphalum* among different individuals of its hosts [60,61,62,63]. The statistical analyses were conducted with SPSS 26.0 and R software (Version 4.3.3). The formulae of Iwao’s regression model and Taylor’s power law were as follows [64,65,66]. The spatial distribution pattern is used to determine the distributing style of a certain population among the sampling units, which is an important issue in ecology. The spatial distribution patterns are usually divided into three types: uniform distribution, random distribution, and aggregated distribution [61,66,67,68].

(5) $$\mathrm{Iwao}’s\mathrm{regression}\mathrm{model}:\;M^{*}=\alpha +\beta M$$

(6) $$M_{i}^{*}=M_{i}+\frac{\sigma_{i}^{2}}{M_{i}}-1$$

(7) $$M_{i}=\frac{\sum_{j=1}^{N_{i}}M_{ij}}{N_{i}}$$

(8) $$\mathrm{Taylor}’s\mathrm{power}\mathrm{law}:\;lg\sigma^{2}=lga+blgm$$

In above formulae, *M_ij_* represents the number of *L. imphalum* on host individual *j* in sampling unit *i*, *N_i_* represents the number of host individuals in sampling unit *i*, and *M_i_* and *σ_i_*^2^ represent the mean and variance of *L. imphalum* on all host individuals in sample unit *i*. M* represents the Lloyd mean crowding, and *M* represents the mean of *L. imphalum* in all sample units; *α* stands for the intercept and *β* the slope in establishing Iwao’s regression model. In Iwao’s regression model, when *α* = 0 and *β* = 1, the spatial distribution pattern was determined to be the random distribution, and when *α* > 0 and *β* > 1, it was the aggregated distribution. In Taylor’s power law, *σ* and *m* (*m = M*) express the same in above formula, *lga* = intercept (the intercept on the *Y*-axis, where *Y* = $lg\sigma^{2}$) and *b* = slope (regression coefficient). When *lga* > 0, *b* = 1, the spatial distribution pattern was determined to be the random distribution, and when *lga* > 0, *b* > 1, it was the aggregated distribution.

### 2.5. Relationship of Zoonotic Diseases to Prevalence of L. imphalum

Based on the documented human cases of two zoonotic diseases, scrub typhus and hemorrhagic fever with renal syndrome (HFRS), in Yunnan Province of southwest China between 2007 and 2015 [69,70,71], the linear regression model was used to analyze the relationship between the infestation prevalence (*P_M_*) of *L. imphalum* on small mammal hosts and the human cases of two zoonotic diseases. The human cases of scrub typhus and HFRS in other four provincial regions (Guizhou, Sichuan, Chongqing, and Tibet) of southwest China, however, were unavailable, and the linear regression analysis did not cover these four provincial regions. In the linear regression analysis, the prevalence (*P_M_*) of *L. imphalum* was used as an explanatory variable (independent variable), and the number of human cases infected with the diseases (scrub typhus or HFRS) was used as a response variable (dependent variable). Since the data did not conform to normal distribution, the “MASS” package in R software (Version 4.3.3) was used for normal transformation [72]. The linear regression analysis was performed with the package “ggpubr” of R software (Version 4.3.3) [73].

### 2.6. Analysis of Influencing Factors on L. imphalum

Six environmental factors (temperature, humidity, elevation, precipitation, landscape, and habitat) were used as the independent variables, and the annual infestation prevalence (*P_M_*) of *L. imphalum* on small mammal hosts was taken as the dependent variable. The analysis of geographical detector (GD) was used to calculate the determination powers of the independent variables (environmental factors) to the dependent variable (*P_M_* of *L. imphalum*). The higher the value of determination power, the more prominent the influence of corresponding factors on the mite (*L. imphalum*). The analysis was conducted with package “GD” in R software (Version 4.3.3) [74,75,76]. The climate factors such as temperature, humidity, and precipitation were obtained from the National Earth System Science Data Center of China (https://www.geodata.cn/main/, accessed on 15 September 2023).

## 3. Results

### 3.1. Collection Sites of L. imphalum

The field investigations were carried out at 114 survey sites in the five provincial regions of southwest China between 2001 and 2022. The names of 114 survey sites and their abbreviations were listed in “Table 1”. A total of 2161 *L. imphalum* were collected from 21 of 114 survey sites (Figure 1). Of 21 sites where *L. imphalum* was collected, 15 sites were distributed in Yunnan Province, 5 sites in the south of Sichuan Province, and 1 site in the southeast of Guizhou Province. There were no *L. imphalum* collected in the other two provincial regions, Chongqing and Tibet (Table 1, Figure 1).

**Table 1:** The 114 survey sites and their abbreviations in southwest China (2001–2022).

No.	Names of Survey Sites and Their Abbreviations		No.	Names of Survey Sites and Their Abbreviations		No.	Names of Survey Sites and Their Abbreviations	
	Names	Abbrs		Names	Abbrs		Names	Abbrs
1	An Yue	AY	39	Jin Sha	JS	77	Se Ni	SN1
2	Ba Yi	BY	40	Jin Tang	JT	78	Shan Nan	SN3
3	Bin Chuan	BC	41	Jiu Long	JL	79	Shong Zhong	SZ1
4	Bo Mi	BM	42	Ka Ruo	KR	80	Shi Zhu	SZ2
5	Cang Yuan	CY1 *	43	Lang Zhong	LZ2	81	Si Mao	SM *
6	Chang Shou	CS	44	Lan Ping	LP1	82	Si Nan	SN2
7	Cha Yu	CY2	45	Le Zhi	LZ1	83	Song Pan	SP
8	Da Fang	DF	46	Liang He	LH1	84	Sui Jiang	SJ
9	Da Li	DL *	47	Li Ping	LP2	85	Teng Chong	TC
10	Dao Cheng	DC2	48	Long Chuan	LC *	86	Tong Nan	TN
11	Da Ying	DY1	49	Long Li	LL2	87	Tong Zi	TZ
12	De Chang	DC1 *	50	Lu Huo	LH2	88	Wan Zhou	WZ
13	De Qin	DQ1	51	Lu Liang	LL1	89	Wei Ning	WN
14	Dian Jiang	DJ	52	Lu Shui	LS	90	Wei Xi	WX *
15	Ding Qing	DQ2	53	Lu Xiang	LX	91	Wei Yuan	WY
16	Du Yun	DY2	54	Ma Er Kang	MEK	92	Wen Shan	WS2
17	Fu Cheng	FC	55	Ma Guan	MG	93	Wu Sheng	WS3
18	Fu Gong	FG	56	Mang Kang	MK	94	Xiang Cheng	XC
19	Fu Ling	FL	57	Mei Tan	MT	95	Xiang Ge Li La	XGLL
20	Fu Yuan	FY *	58	Meng Hai	MH *	96	Xi Xiu	XX
21	Gan Zi	GZ	59	Meng La	ML3 *	97	Xuan Han	XH
22	Geng Ma	GM *	60	Meng Zi	MZ	98	Xu Zhou	XZ
23	Gong Bu Jiang Da	GBJD	61	Mian Ning	MN	99	Ya Jiang	YJ3
24	Gong Shan	GS	62	Mi Lin	ML1	100	Yan Bian	YB2
25	Guan Ling	GL	63	Mu Yi	MY	101	Yan Yuan	YY1 *
26	Gui Ding	GD	64	Mu Li	ML2 *	102	Ying Jiang	YJ2
27	He Kou	HK	65	Ning Er	NE *	103	Yong De	YD
28	Hong Ya	HY1	66	Ping Chang	PC	104	You Yang	YY3
29	Hong Yuan	HY2	67	Ping Shan	PS	105	Yuan Jiang	YJ1 *
30	Hua Xi	HX	68	Pu An	PA	106	Yu Long	YL *
31	Hui Dong	HD *	69	Jian Wei	JW	107	Yun Yang	YY2
32	Hui Shui	HS	70	Qiao Jia	QJ	108	Zhao Jue	ZJ1 *
33	Jian Chuan	JC *	71	Qiu Bei	QB	109	Zhen Feng	ZF
34	Jiang Jin	JJ	72	Ren Shou	RS	110	Zheng An	ZA
35	Jiang Kou	JK	73	Rong Jiang	RJ *	111	Zhi Jin	ZJ2
36	Jiang Yang	JY	74	Rui Li	RL *	112	Zhong Shan	ZS
37	Jing Hong	JH *	75	Ruo Er Gai	REG	113	Zhong Xian	ZX
38	Jin Ping	JP	76	San Sui	SS	114	Zi Zhong	ZZ

Annotation: The survey sites marked with “*” were the sites where *Leptotrombidium imphalum* was collected.

### 3.2. Hosts of L. imphalum

**Figure 2:**
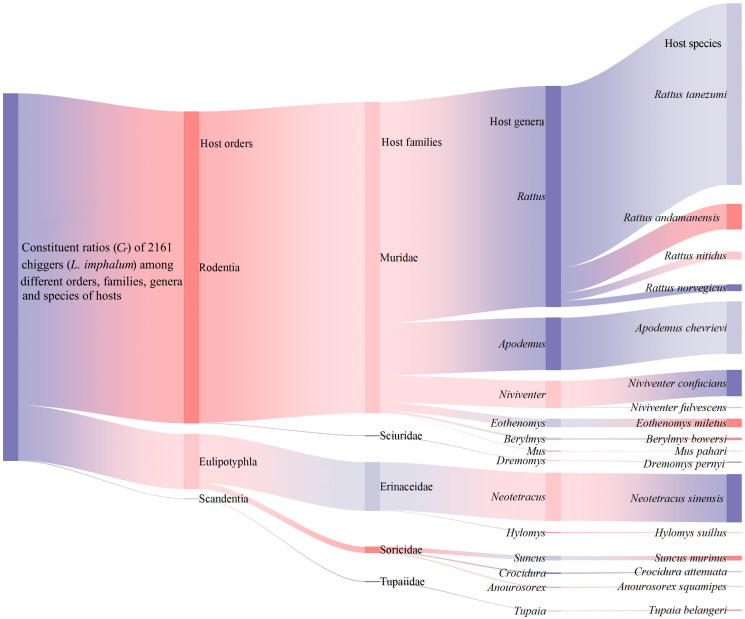
The visualization of the constituent ratios (*C_r_*) of 2161 chiggers (*L. imphalum*) among different orders, families, genera, and species of hosts (218 small mammal hosts) in southwest China (2001–2022). Annotation: The shade width indicates the constituent ratio of the chigger *L. imphalum* on a certain order, family, genus, or species of the host.

The 2161 individuals of *L. imphalum* were identified from 218 small mammal hosts, which belong to 17 species, 13 genera, and 5 families in 3 orders, Rodentia, Eulipotyphla, and Scandentia, in which the dominant host species was the Asian house rat or Oriental house rat, *Rattus tanezumi* (the constituent ratio *C_r_* = 40.83%, 89/218) in the genus *Rattus*, family Muridae, and order Rodentia (Table 2). At the order level of hosts, the majority of *L. imphalum* (*C_r_* = 84.87%, 1834/2161) came from the order Rodentia (rodents), which were the most important hosts of *L. imphalum*. The number of *L. imphalum* from the order Eulipotyphla (insectivores) came next with *C_r_* = 14.90% (322/2161), and only five mites (*C_r_* = 0.23%, 5/2161) were identified from the order Scandentia (tree shrews). At the species level of hosts, 49.38% of *L. imphalum* (*C_r_* = 49.38%, 1067/2161) were identified from *R. tanezumi*, and “Figure 2” visualized in detail the constituent ratios (*C_r_*) of the identified 2161 *L. imphalum* among different orders, families, genera, and species of the hosts, 218 small mammals. The infestation indices of *L. imphalum* varied among different host species, with the highest prevalence (*P_M_* = 27.66%), mean abundance (*MA* = 6 mites/per examined host), and mean intensity (*MI* = 21.69 mites/per infested host) on the shrew gymnure, *Neotetracus sinensis* (Table 2) (*p* < 0.05).

### 3.3. Infestation Variations of Leptotrombidium imphalum in Different Environments

To analyze infestation variations of *L. imphalum* on its small mammal hosts in different environments, the survey sites with *L. imphalum* collected were categorized as different landscapes (mountainous and flatland landscapes), different habitats (indoor and outdoor habitats), and different altitude gradients (500–1000 m, 1001–1500 m, 1501–2000 m, 2001–2500 m, and 2501–3000 m). The results showed that the infestation indices for *L. imphalum* on small mammal hosts varied in different environments. The differences of the infestation indices along different altitude and latitude gradients were statistically significant (*p* < 0.05) (Table 3 and Table 4), but those in different landscapes (mountainous and flatland landscapes) and different habitats (indoor and outdoor habitats) were not statistically significant (*p* > 0.05) (Table 5 and Table 6).

### 3.4. Spatial Distribution Pattern of L. imphalum

Iwao’s regression model and Taylor’s power law were used to analyze the spatial distribution pattern of *L. imphalum* among different individuals of its small mammal hosts. The calculated Iwao’s regression formula was M* = 10.44 + 2.88*M* (R^2^ = 0.86, *p* < 0.05), with both *α* and *β* (*α* = 10.44, *β* = 2.88) exceeding boundary values (0 and 1) for determining the aggregated distribution. The calculated Taylor’s power formula was $lg\sigma^{2}=0.65+1.7lgm$ (R^2^ = 0.91, *p* < 0.05), with both *lga* and *b* (*lga* = 0.65, *b* = 1.7) also exceeding the boundary values (0 and 1) for determining the aggregated distribution.

### 3.5. Relationship of Zoonotic Diseases to Prevalence of L. imphalum

**Figure 3:**
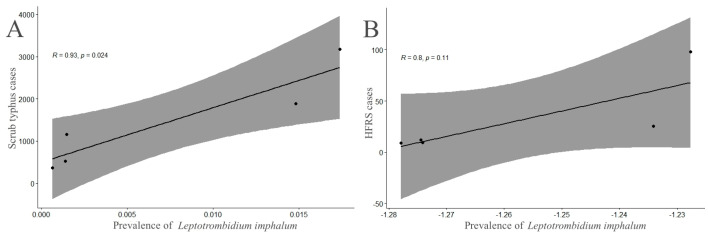
Linear correlations between the prevalence of *Leptotrombidium imphalum* on small mammals and the documented human cases of two zoonotic diseases, scrub typhus (**A**) and HFRS (**B**), in Yunnan Province of southwest China (2007–2015). The grey zone is the 95% confidence interval, and the dark line is the linear regression line. (Annotation: (**B**) in the above figure were those after normal transformation due to the fact that the data did not conform to normal distribution).

Table 7 listed the documented human cases of two zoonotic diseases (scrub typhus and hemorrhagic fever with renal syndrome, HFRS) from Yunnan Province of southwest China between 2007 and 2015 [69,70,71]. The results of the linear regression analysis showed that the infestation prevalence (*P_M_*) of *L. imphalum* on small mammals had high explanatory power for the incidence of scrub typhus (R = 0.93, *p* < 0.05) but not for the incidence of HFRS (R = 0.8, *p* > 0.05) (Figure 3).

**Table 7:** The human cases of scrub typhus and hemorrhagic fever with renal syndrome (HFRS) and the infestation prevalence of *L. imphalum* on small mammals in Yunnan Province of southwest China (2007–2015).

Year	Scrub Typhus Cases	HFRS Cases	Prevalence (*P_M_*, %) of *L. imphalum*
2007	365	14	0.06
2008	526	20	0.14
2010	1155	15	0.15
2012	1884	49	1.48
2015	3176	264	1.74

### 3.6. Analysis of Influencing Factors on L. imphalum

Of six environmental factors (temperature, humidity, elevation, precipitation, landscape, and habitat), the determination powers of humidity and temperature (independent variables) to the *P_M_* of *L. imphalum* (dependent variable) reached 69.77% (*p* < 0.01) and 67.02% (*p* < 0.01), respectively. The determination power of the mutual interaction of these two factors (humidity and temperature) went up to 96.26% (*p* < 0.05).

## 4. Discussion

As mentioned in “Section 1”, the most confirmed vectors of scrub typhus are concentrated in the genus *Leptotrombidium*, and from this point of view, most chigger species of *Leptotrombidium* have the potential to transmit the disease, though there has been no direct evidence for many *Leptotrombidium* species to date [14,20,21,22]. In China, six chigger species in the genus *Leptotrombidium* are the main vectors of scrub typhus, and they are *L. deliense* (Walch, 1922); *L. scutellare* Nagayo et al., 1921; *L. rubellum* Wang et Liao, 1984; *L. wenense* Wu et al., 1982; *L. insulare* Wei et al., 1989; and *L. sialkotense* Vercammen-Grandjean and Langston, 1976 [8,77,78]. Additionally, *L. scutellare* has also been identified as a potential vector of hemorrhagic fever with renal syndrome (HFRS) in China [7,8]. Besides the six main vectors of scrub typhus, *L. imphalum* and some other chigger species (more than ten species) are also important potential vectors of the disease in China [36,79,80,81,82]. Numerous pieces of evidence have demonstrated that *L. imphalum* can be an effective vector of scrub typhus [27,28,29,30]. For example, the pathogen of scrub typhus (*O. tsutusgamushi*, Ot) has been isolated from *L. imphalum* in Yunnan Province of southwest China [30,31]. The transstadial and transovarial transmission of *O. tsutusgamushi* in *L. imphalum* have been achieved in the laboratory, and the temporal changes in prevalence of *O. tsutsugamushi* infecting the eggs of *L. imphalum* also support the disease transmission efficiency of the mite [27,29,33,34,35,83]. Although *L. imphalum* is medically important, few studies have involved the distribution, infestation, and related ecology of this vector species on rodents and other small mammal hosts in a vast geographical territory like southwest China. For the first time, this paper reported the distribution and infestation status of *L. imphalum* on its small mammal hosts in southwest China, an important focus of scrub typhus and HFRS [9,10,11,79,84].

The results of the present study showed that 2161 *L. imphalum* were identified from 17 species of small mammal hosts in 21 of 114 survey sites in southwest China, and the 17 host species crossed 13 genera and 5 families in 3 orders (Rodentia, Eulipotyphla, and Scandentia), indicating the low host specificity of the mite. Previous studies have demonstrated that most chigger species have low host specificity, which is beneficial to their transmission of scrub typhus among different animal hosts [54,85,86]. The low host specificity of *L. imphalum* may increase the potential risk of the mite. Although the host specificity of *L. imphalum* is low, the mite still has a preference for some specific hosts. In the present study, the majority of *L. imphalum* were identified from rodents (the order Rodentia), indicating that rodents are the main hosts of *L. imphalum* in southwest China. The infestation indices for *L. imphalum* varied among different species of small mammal hosts, with the highest *P_M_*, *MA*, and *MI* on the shrew gymnure, *N. sinensis* (*p* < 0.05), indicating that *N. sinensis* is more susceptible to the infestation of *L. imphalum*. In the present study, the infestation indices for *L. imphalum* on small mammal hosts varied along different altitude and latitude gradients (*p* < 0.05), and this result reveals the environmental heterogeneity of chigger infestations [86,87]. The result of the geographical detector (GD) analysis showed that the determination power of the mutual interaction of temperature and humidity to the infestation prevalence (*P_M_*) of *L. imphalum* on small mammals was as high as 96.26% (*p* < 0.05). This result suggested that temperature and humidity can significantly influence the infestation of *L. imphalum*. The results of the present study are consistent with previous studies [51,86,88].

The spatial distribution pattern in the present study refers to the distribution of *L. imphalum* among different individuals of its small mammal hosts. In ecology, the spatial distribution patterns are usually divided into three types: uniform distribution, random distribution, and aggregated distribution [66,67,68]. The results of the present study revealed that the values of *α* and *β* (*α* = 10.44, *β* = 2.88) in Iwao’s regression analysis and the values of *lga* and *b* (*lga* = 0.65, *b* = 1.7) in Taylor’s power law all exceeded the boundary values (0 and 1) for determining the aggregated distribution. Therefore, the spatial distribution pattern of *L. imphalum* among different individuals of small mammal hosts were determined to be of an aggregated distribution. This aggregated distribution means that chiggers (*L. imphalum*) are not evenly distributed among different host individuals. Some hosts have few or no chiggers on their body surface, while other hosts have many chiggers, forming a mite colony [89,90,91]. The aggregated distribution of *L. imphalum* is consistent with previous reports on some other species of chigger mites, and this aggregated distribution pattern may be beneficial to the growth, development, and reproduction of chigger mites and other parasites [53,92,93,94]. The results of the linear regression analysis in the present study showed that the prevalence (*P_M_*) of *L. imphalum* had high explanatory power for the incidence of scrub typhus, with a high positive correlation between the *P_M_* of *L. imphalum* and the human cases of scrub typhus (R = 0.93, *p* < 0.05), but not for the incidence of HFRS (Figure 3). The results further suggest that *L. imphalum* is closely related to the prevalence of scrub typhus but not related to that of HFRS. Although *L. imphalum* has been identified as an important vector of scrub typhus, there has been no evidence showing that it can serve as a vector of HFRS and other zoonotic diseases [26,33,95]. The investigated region (southwest China) of the present study is one of main foci of scrub typhus in China [11,84,96], the positive correlation between the *P_M_* of *L. imphalum* and the human cases of scrub typhus, together with the low host specificity of the mite, may increase the potential risk of the mite’s transmission of scrub typhus in southwest China.

## Figures and Tables

**Table 2:** The constituent ratios and infestation indices of *Leptotrombidium imphalum* on different small mammal hosts in southwest China (2001–2022).

Host Species	No. of Hosts	No. of *L. imphalum* and Its Constituent Ratio (*C_r_*, %)		Infestation Indices of *L. imphalum*		
		Number	*C_r_*, %	Prevalence (*P_M_*, %)	Mean Abundance (*MA*)	Mean Intensity (*MI*)
*Rattus tanezumi*	89	1067	49.38	3.88 *	0.46 *	11.99 *
*Apodemus chevrieri*	29	308	14.25	2.36 *	0.25 *	10.62 *
*Neotetracus sinensis*	13	282	13.05	27.66 *	6 *	21.69 *
Other species	87	504	23.32	1.57 *	0.09 *	5.79 *
Total	218	2161	100			

Annotation: The symbol “*” represents *p* < 0.05.

**Table 3:** Infestation variations of *Leptotrombidium imphalum* on small mammal hosts along different altitude gradients in southwest China (2001–2022).

Altitudes Gradients (m)	No. of Hosts	No. and Constituent Ratios of *L. imphalum*		Infestation Indices of *L. imphalum*		
		No.	*C_r_*, %	*P_M_*, %	*MA*	*MI*
500–1000	76	694	32.11	4.68 *	0.43 *	9.13 *
1001–1500	41	423	19.58	3.92 *	0.40 *	10.32 *
1501–2000	56	759	35.12	3.06 *	0.41 *	13.55 *
2001–2500	42	282	13.05	1.20 *	0.07 *	6.71 *
2501–3000	3	3	0.14	0.43 *	0.00 *	1 *
Total	218	2161	100			

Annotation: The confidence intervals are 99.9–100% for the prevalence (*P_M_*), 98.8–99.8% for the mean abundance (*MA*), and 94.1–99.0% for the mean intensity (*MI*), respectively. The symbol “*” represents *p* < 0.05.

**Table 4:** Infestation indices of *Leptotrombidium imphalum* in different latitude gradients.

Latitude (°N)	No. of Hosts	No. and Constituent Ratios of *Leptotrombidium imphalum*		Infestation Indices of *Leptotrombidium imphalum*		
		Number	*C_r_*, %	*P_M_*, %	*MA*	*MI*
21–22	101	1023	47.34	10.69 *	1.08 *	10.13 *
23–24	19	53	2.45	1.04 *	0.03 *	2.79 *
25–26	71	814	37.67	1.30 *	0.15 *	11.46 *
27–28	27	271	12.54	1.24 *	0.12 *	10.04 *
Total	218	2161	100			

Annotation: The confidence intervals are 99.9–100% for *P_M_*, 98.5–99.7% for *MA*, and 98.3–100% for *MI*, respectively. The symbol “*” represents *p* < 0.05.

**Table 5:** Infestation indices of *Leptotrombidium imphalum* in different habitats.

Habitats	No. of Hosts	No. and Constituent Ratios of *Leptotrombidium imphalum*		Infestation Indices of *Leptotrombidium imphalum*		
		Number	*C_r_*, %	*P_M_*, %	*MA*	*MI*
Indoor	4	10	0.46	0.69	0.02	2.5
Outdoor	214	2151	99.54	2.15	0.22	10.05
Total	218	2161	100			

Annotation: The confidence intervals are 99.0–100% for *P_M_*, 97.0–99.5% for *MA*, and 98.3–100% for *MI*, respectively.

**Table 6:** Infestation indices of *Leptotrombidium imphalum* in different landscapes.

Landscapes	No. of Hosts	No. and Constituent Ratios of *Leptotrombidium imphalum*		Infestation Indices of *Leptotrombidium imphalum*		
		Number	*C_r_*, %	*P_M_*, %	*MA*	*MI*
Mountainous	151	1445	66.87	2.03	0.19	9.57
Flatland	67	716	33.13	2.18	0.23	10.69
Total	218	2161	100			

Annotation: The confidence intervals are 99.9–100% for *P_M_*, 99.5–99.9% for *MA*, and 98.3–100% for *MI*, respectively.

## Data Availability

The experimental data used to support the findings of this study are available from the corresponding author request.
